# *Mycobacterium tuberculosis* Acquires Limited Genetic Diversity in Prolonged Infections, Reactivations and Transmissions Involving Multiple Hosts

**DOI:** 10.3389/fmicb.2017.02661

**Published:** 2018-01-19

**Authors:** Marta Herranz, Ilva Pole, Iveta Ozere, Álvaro Chiner-Oms, Miguel Martínez-Lirola, Felipe Pérez-García, Paloma Gijón, María Jesús Ruiz Serrano, Laura Clotet Romero, Oscar Cuevas, Iñaki Comas, Emilio Bouza, Laura Pérez-Lago, Darío García-de-Viedma

**Affiliations:** ^1^Servicio Microbiología Clínica y Enfermedades Infecciosas, Hospital General Universitario Gregorio Marañón, Madrid, Spain; ^2^Instituto de Investigación Sanitaria Gregorio Marañón, Madrid, Spain; ^3^CIBER Enfermedades Respiratorias (CIBERES), Madrid, Spain; ^4^Childhood Tuberculosis Department, Centre of Tuberculosis and Lung Diseases, Riga East University Hospital, Riga, Latvia; ^5^Latvian Biomedical Research and Study Centre, Riga, Latvia; ^6^Department of Infectology and Dermatology, Riga Stradinš University, Riga, Latvia; ^7^Unidad Mixta Genómica y Salud, Centro Superior de Investigación en Salud Pública (FISABIO)-Universitat de València, Valencia, Spain; ^8^Servicio de Microbiología, Complejo Hospitalario Torrecárdenas, Almería, Spain; ^9^Servei de Vigilància Epidemiològica i Resposta a Emergències de Salut Pública al Vallès Occidental i Vallès Oriental, Subdirecció General de Vigilància i Resposta a Emergències de Salut Pública, Agència de Salut Pública de Catalunya, Barcelona, Spain; ^10^Servicio de Laboratorio, Institut d'Investigació i Innovació Parc Taulí, I3PT Parc Taulí Hospital Universitari, Universitat Autònoma de Barcelona, Barcelona, Spain; ^11^Instituto de Biomedicina de Valencia, Consejo Superior de Investigaciones Científicas, Valencia, Spain; ^12^CIBER en Epidemiología y Salud Pública, Madrid, Spain; ^13^Departamento de Medicina, Facultad de Medicina, Universidad Complutense de Madrid, Madrid, Spain

**Keywords:** tuberculosis, variability, whole genome sequencing, SNPs, microevolution

## Abstract

**Background:** *Mycobacterium tuberculosis* (MTB) has limited ability to acquire variability. Analysis of its microevolution might help us to evaluate the pathways followed to acquire greater infective success. Whole-genome sequencing (WGS) in the analysis of the transmission of MTB has elucidated the magnitude of variability in MTB. Analysis of transmission currently depends on the identification of clusters, according to the threshold of variability (<5 SNPs) between isolates.

**Objective:** We evaluated whether the acquisition of variability in MTB, was more frequent in situations which could favor it, namely intrapatient, prolonged infections or reactivations and interpatient transmissions involving multiple sequential hosts.

**Methods:** We used WGS to analyze the accumulation of variability in sequential isolates from prolonged infections or translations from latency to reactivation. We then measured microevolution in transmission clusters with prolonged transmission time, high number of involved cases, simultaneous involvement of latency and active transmission.

**Results:** Intrapatient and interpatient acquisition of variability was limited, within the ranges expected according to the thresholds of variability proposed, even though bursts of variability were observed.

**Conclusions:** The thresholds of variability proposed for MTB seem to be valid in most circumstances, including those theoretically favoring acquisition of variability. Our data point to multifactorial modulation of microevolution, although further studies are necessary to elucidate the factors underlying this modulation.

## Introduction

Whole-genome sequencing (WGS) has transformed the way we analyze transmission of tuberculosis (TB) (Nikolayevskyy et al., 2016; Comas, 2017; Satta et al., 2017). Identification of transmission clusters is currently based on determination of the magnitude of genomic diversity among the isolates in a population. An exhaustive analysis of the magnitude of this variability acquired in different clinical/epidemiological situations led to the definition of thresholds to determine whether 2 isolates were part of the same transmission chain, i.e., clustered (<5 SNPs) or unrelated (>12 SNPs) (Walker et al., 2013). Since then, these thresholds have been used as a consensus reference, and it has been accepted that microevolution in *Mycobacterium tuberculosis* (MTB) infection occurs within those ranges and with no wider deviations expected (Casali et al., 2016; Seto et al., 2017; Witney et al., 2017).

Acquisition of variability in MTB has been studied not only to determine the SNP thresholds to be applied in genomic epidemiology, but also to help us to understand one of the microevolution paths in MTB infection which may lead to more successful variants, in addition to other molecular events such as insertion/deletions or intragenomic recombination.

Different studies have addressed the emergence of clonal variants in MTB infections according to different molecular markers (Warren et al., 2002; Shamputa et al., 2006; Al-Hajoj et al., 2010; Navarro et al., 2011; Black et al., 2015). The magnitude of intrapatient diversity which can be measured for a strain within the same patient has been found to be comparable to the one observed after sequential rounds of multiple hosts sequentially along transmission chains (Pérez-Lago et al., 2014). Sometimes, these variants have been found associated to prolonged infections or to a history of previous TB (Navarro et al., 2011), suggesting that infection time longer than the average and reactivation events could be associated with the accumulation of genetic diversity. The characteristics of the healthcare system, the efficiency of the drug treatment and the socioeconomic status also have a role on the genetic diversity expected. The analysis of diversity in the population of MTB has been found to be associated in certain cases with substandard life conditions, delayed diagnosis and improper therapeutical management and poor adherence to treatment, factors which can be responsible for prolonged infections (Shamputa et al., 2006; Navarro et al., 2011).

Post-mortem genetic analysis of the MTB population within individuals (Cohen et al., 2011) has demonstrated the accumulation of diversity by MTB after a prolonged infection period. It has been shown with special exhaustivity in a recent article by Lieberman et al. (2016), based on post-mortem multiple sampling and WGS analysis in patients who had received only minimal anti-TB treatment.

The identification of genetic diversity is not a meaningless finding and different authors have found an effect of certain subtle genetic modifications and the expression of neighboring genes and even in the infectivity between closely related variants and the potential functional role of microevolution has been explored (Soto et al., 2004; Tantivitayakul et al., 2010; Pérez-Lago et al., 2011, 2013).

Therefore, although the similarity thresholds defined in the genomic epidemiology of TB (Walker et al., 2013) are based on a wide and solid analysis of patients, additional analysis of microevolution in MTB in specific clinical/epidemiological situations that might represent greater opportunities to acquire variability, which were not extensively represented in the studies defining these cutoffs, could help us to evaluate the robustness of the similarity thresholds proposed and to understand the influence of various infection scenarios on microevolution dynamics.

## Materials and methods

### Genotyping by MIRU-VNTR analysis

24-Locus MIRU-VNTR multiplex analysis was performed from cultured isolates. DNA was purified using the Qiagen DNA MiniKit (Qiagen, Hilden, Germany).

The final reaction mixture (50 μl) included 25 μl of PCR Master Mix (QIAGEN multiplex PCR kit), 5 μl of Q solution, and 0.25 μM of each labeled and unlabeled oligonucleotide (3.9 μM for loci 4156 and 2059). The primers used for PCR amplification and PCR conditions have been reported elsewhere (Supply et al., 2006; Oelemann et al., 2007). PCR products were sized using capillary electrophoresis in an ABI Prism 3100 genetic analyzer (Applied Biosystems, NLLab Centraal B.V., Haarlem, The Netherlands). MIRU-VNTR types were compared using Bionumerics (4.6 Applied Maths, Sint-Martens-Latem, Belgium).

### Whole-genome analysis

DNA was extracted, after recovering all the bacteria present in the liquid cultures by centrifugation, using the standard cetyl trimethyl ammonium bromide (CTAB) method, and DNA libraries were generated following the Nextera XT Illumina protocol (Nextera XT Library Prep kit [FC-131-1024]). Library quality and size distribution were checked by running 2 μl on a 2200 TapeStation Bioanalyzer (Agilent Technologies, USA). The libraries were then normalized based on the average fragment size observed, the library concentration was measured using Qubit 2.0 Fluorometer (Life Technologies, US), and the libraries were pooled. Paired-end sequences were obtained using a MiSeq platform, with an average per base coverage of 87x (range 62x−113).

SNP calling was performed as indicated elsewhere (Pérez-Lago et al., 2014). In summary, after mapping to a hypothetical MTB ancestral genome (identical to H37Rv according to structure but including the maximum-likelihood-inferred ancestral nucleotide positions from a virtual ancestor; Comas et al., 2010), we extracted all variable positions in the strain of interest. In order to avoid false-positive calls, a series of quality filters were applied to data associated with the SNP. First of all, a minimum coverage >20x and mapping quality 20 were required. From all the variants detected, we divide them into homozygous (present at least in 90% of the reads) and heterozygous calls (present in less than 90% of the reads). Only those SNPs in heterozygosis that appear in homozygosis in other member of the cluster, were selected to be included in the analyses. False positive variants could appear due to mapping errors in genome repetitive regions or near indels. So, we filter out these potential errors by omitting from our analysis variants detected in repetitive regions, phages and PE/PPE regions. Also, those variants found near indel areas and in regions with an anomalous accumulation of SNPs (3 or more SNPs in a 10 bp window) were omitted.

Alignments and SNP variants were visualized and checked using the IGV program. Multiple comparisons between the SNPs from different isolates were performed using an in-house script written in R.

Fastq files with the raw data for isolates are deposited (http://www.ebi.ac.uk) under accession numbers ERS2016357-ERS2016427 and ERP002297.

The dN/dS ratio was calculated using the total number of synonymous and non-synonymous variants for the intra- and inter-patient groups. The potential synonymous and non-synonymous sites for the *M. tuberculosis* genome were obtained using the SNAP tool (Ota and Nei, 1994). All the regions omitted in the variant calling pipeline (repeats, phages and PE/PPE) were also omitted when calculating the dN/dS ratio. The reference dataset used to compare the dN/dS ratio was obtained from a previously published work (12808 synonymous SNPs and 21118 non-synonymous SNPs; Comas et al., 2013). Fisher's exact test was applied in the comparison of nonsynonimous/synonymous ratio.

The median-joining networks were constructed from the SNP matrix generated for each case using NETWORK 5.0.0.1.

## Results

We aimed to select representative examples to illustrate deviations in the standard infective circumstances faced by MTB that could provide MTB with better opportunities to acquire variability. We examined both intrapatient scenarios (single infections in selected cases) and interpatient scenarios (transmission chains involving serial hosts). We selected 2 settings in which we could expect a more marked intrapatient microevolution: prolonged infections due to poor adherence to treatment and cases in which reactivation occurred sometime after the microbiological cure of the first episode. For the interpatient evaluation, extensive active transmission clusters involving a high number of cases and clusters involving both latency and active transmission events were selected to attempt to obtain a selective pressure that was higher than average.

### Intrapatient analysis: prolonged infections

**Figure 1:**
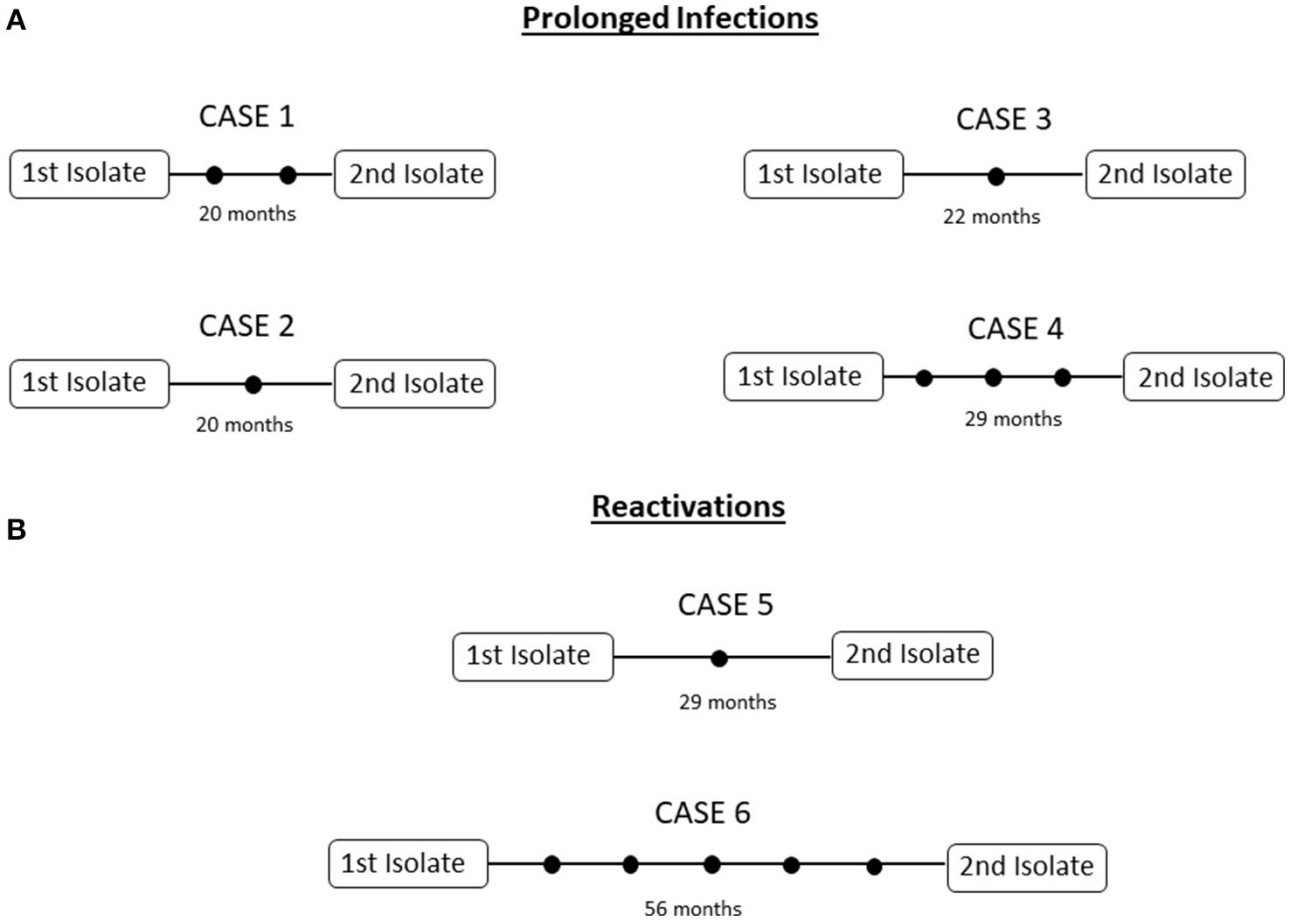
Networks of relationships for the intrapatient **(A)** prolonged infections, **(B)** reactivations in analysis. Each black dot corresponds to a SNP.

We selected 4 cases involving poor adherence to treatment in which 2 sequential MTB isolates (Supplementary Table 1, cases 1–4; all pansusceptible) had been obtained for the same episode over a longer period than usual (20–29 months). Reinfection was excluded because the paired isolates shared identical MIRU-VNTR patterns (data not shown). The comparative WGS analysis of the paired isolates revealed the occurrence of variability, with a range of 1-3 SNPs between isolates (Figure 1A; Supplementary Table 1).

### Intrapatient analysis: reactivations

Two cases with complete adherence to treatment and a second isolate with an identical MIRU-VNTR pattern obtained 27 and 56 months after the resolution of the first episode were selected as representatives of reactivation. We evaluated whether the stress arising from the transition from latency to reactivation could lead to greater accumulation of variability. Microevolution was identified in both cases, and 1 and 5 SNPs were found between the sequential isolates (Supplementary Table 1, cases 5 and 6) (Figure 1B).

### Interpatient analysis: extensive active transmission clusters

**Figure 2:**
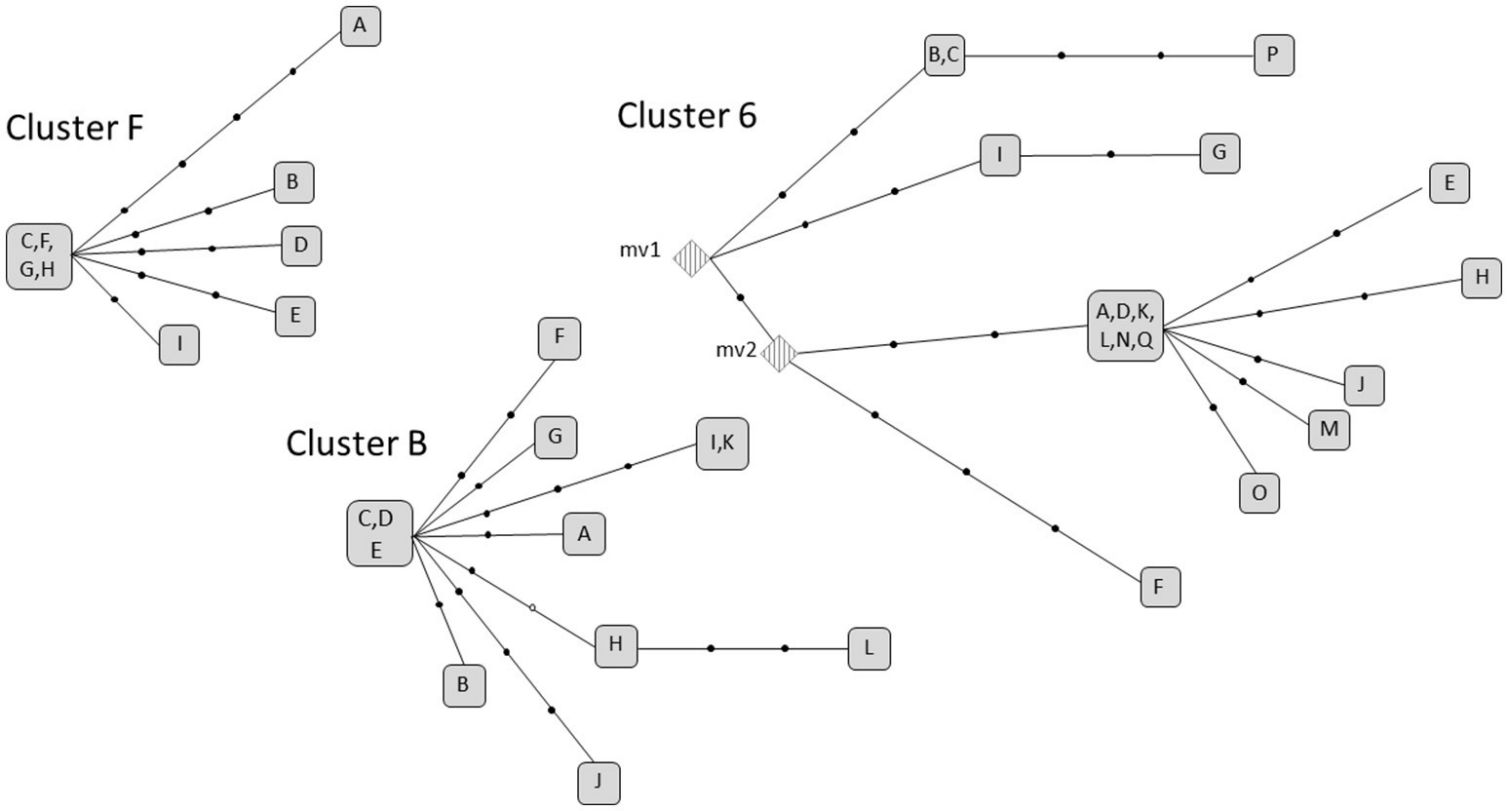
Networks of relationships for the extensive active transmission clusters in analysis. Each black dot corresponds to a SNP. The letters correspond to the isolates. Those sharing the same squared box shared the same polymorphisms. The white dot in Cluster B indicates a heterozygous SNP. mv: (median vector) non-sampled isolates inferred from the distribution of SNPs in the network.

We selected 3 clusters (cluster F, 6 and B) that fulfilled the double criteria of prolonged infection (6, 11, and 12 years, respectively) and a high number of cases (9–17 cases). In all cases, the strains involved were pansusceptible. The microevolution observed in each cluster led to accumulation of, i.e., 11, 20, and 15 SNPs (one of which was in heterozygosis), respectively (Supplementary Tables 2–4). However, the accumulation dynamics of SNPs between each 2 linked cases in the transmission networks was moderate (Figure 2), with the maximum distances between any of 2 clustered isolates in clusters 6, B, and F being 9, 7, and 6.

### Interpatient analysis: clusters including both latency and active transmission

We now aimed to analyze events with coincidental involvement of latency and active transmission through sequential hosts and added the requirements of prolonged observation periods and/or the involvement of a high number of cases. We identified 3 clusters in 3 different geographic settings (Cluster 1 in Madrid, Spain; Cluster 2 in Sabadell, Spain; and Cluster 3 in Latgale, Latvia) fulfilling as many coincidental factors as possible (see above).

**Figure 3:**
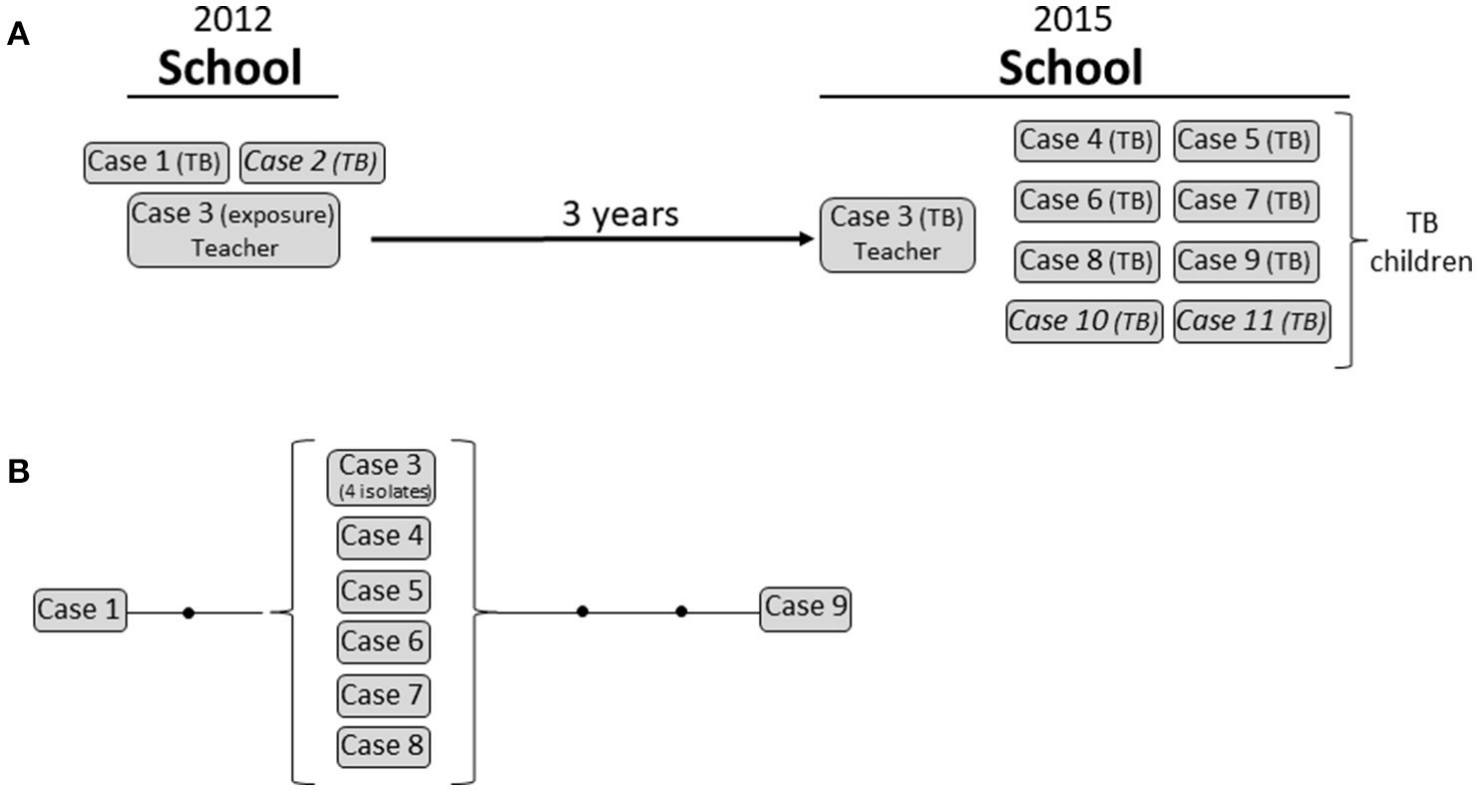
Cluster 1. **(A)** Graphical scheme of the cluster including chronology, transmission settings, and cases involved. **(B)** Network of relationships for the isolates analyzed by WGS. Each black dot corresponds to a SNP. Isolates not available for the WGS analysis are in italic.

Cluster 1 corresponded to an 11-case outbreak in a school in Madrid. The event consisted of a double outbreak involving the same setting but 3 years apart (Figure 3). In 2012, 2 MTB isolates with identical MIRU-VNTR patterns were obtained from 2 epidemiologically linked cases (a child from the school and the father of a child from another class). At the time, contact tracing was incomplete. One of the teachers (case 3)—likely exposed in the 2012 event—developed active TB 3 years later (2015), and 8 new cases of culture-positive TB were diagnosed among the children in the school. All 11 isolates were identical according to the MIRU-VNTR analysis. Unfortunately, 3 isolates were not available for WGS, although we included 4 isolates from the teacher to study intrapatient variability.

We expected that the combination of these coincidental factors (latency, active transmission, and multi-host infection) in this complex event would reveal marked variability between the strains involved. However, global variability in SNPs was limited (Figure 3; Supplementary Table 5). All 4 isolates from the teacher and 5 out 6 isolates from 2015 were identical (0 SNPs) and showed only 1 SNP of difference compared with the 2012 isolate (case 1). One of the isolates from 2015 (case 9) showed 2 additional SNPs.

**Figure 4:**
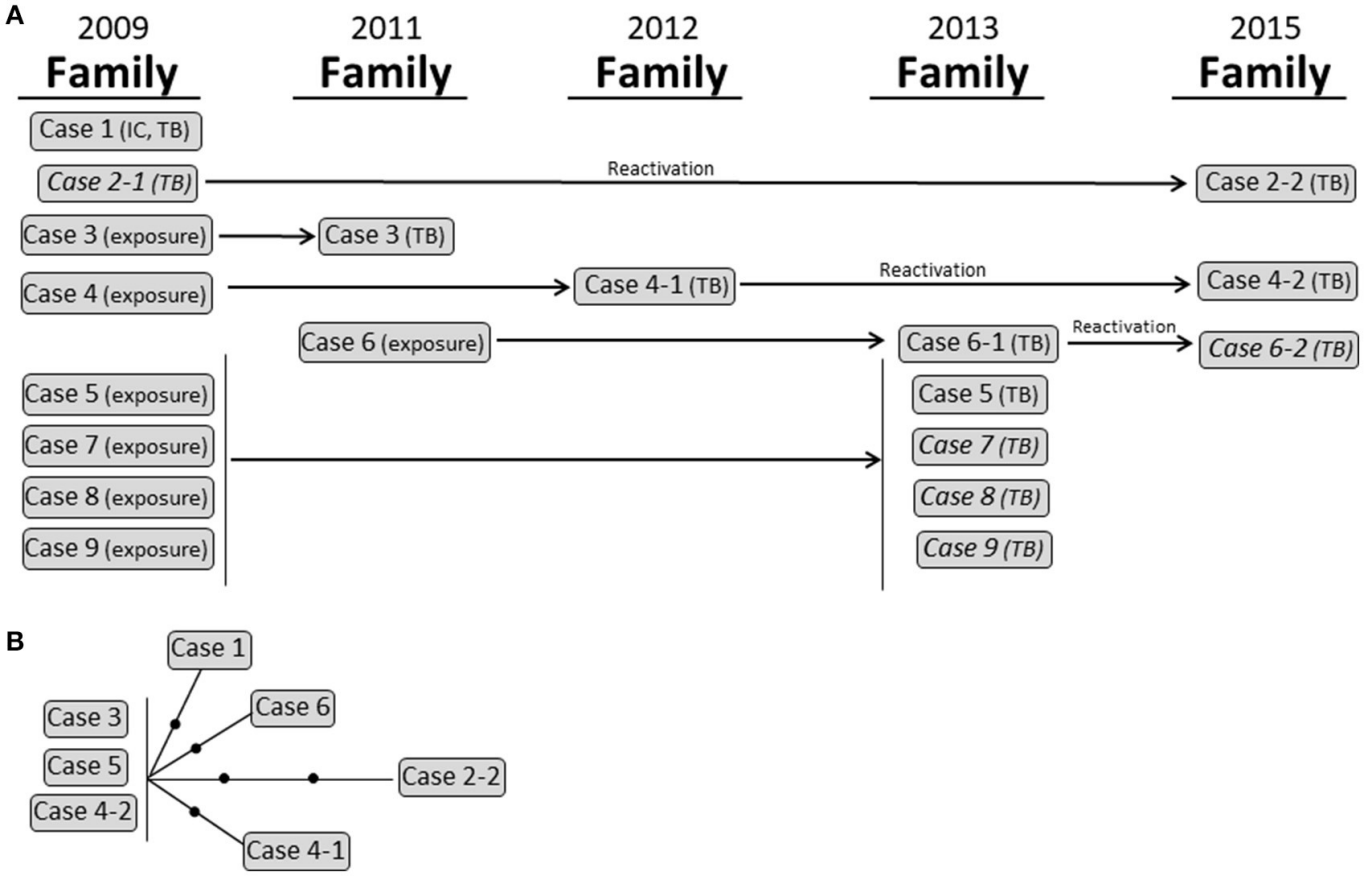
Cluster 2. **(A)** Graphical scheme of the cluster including chronology, transmission settings, and cases involved. **(B)** Network of relationships for the isolates analyzed by WGS. Each black dot corresponds to a SNP. Isolates not available for the WGS analysis are in italic. IC: index case.

Cluster 2 corresponded to a complex family outbreak in Sabadell involving 9 cases and spanning 6 years owing to the overlap of active transmission events and latency periods between the exposure of specific cases where the disease developed some years later and eventually coincided with 3 reactivations (Figure 4). Seven isolates were available for study, and all of them were identical according to MIRU-VNTR analysis. Despite the complexity of the cluster, WGS revealed global variability for only 5 SNPs (Figure 4; Supplementary Table 5).

**Figure 5:**
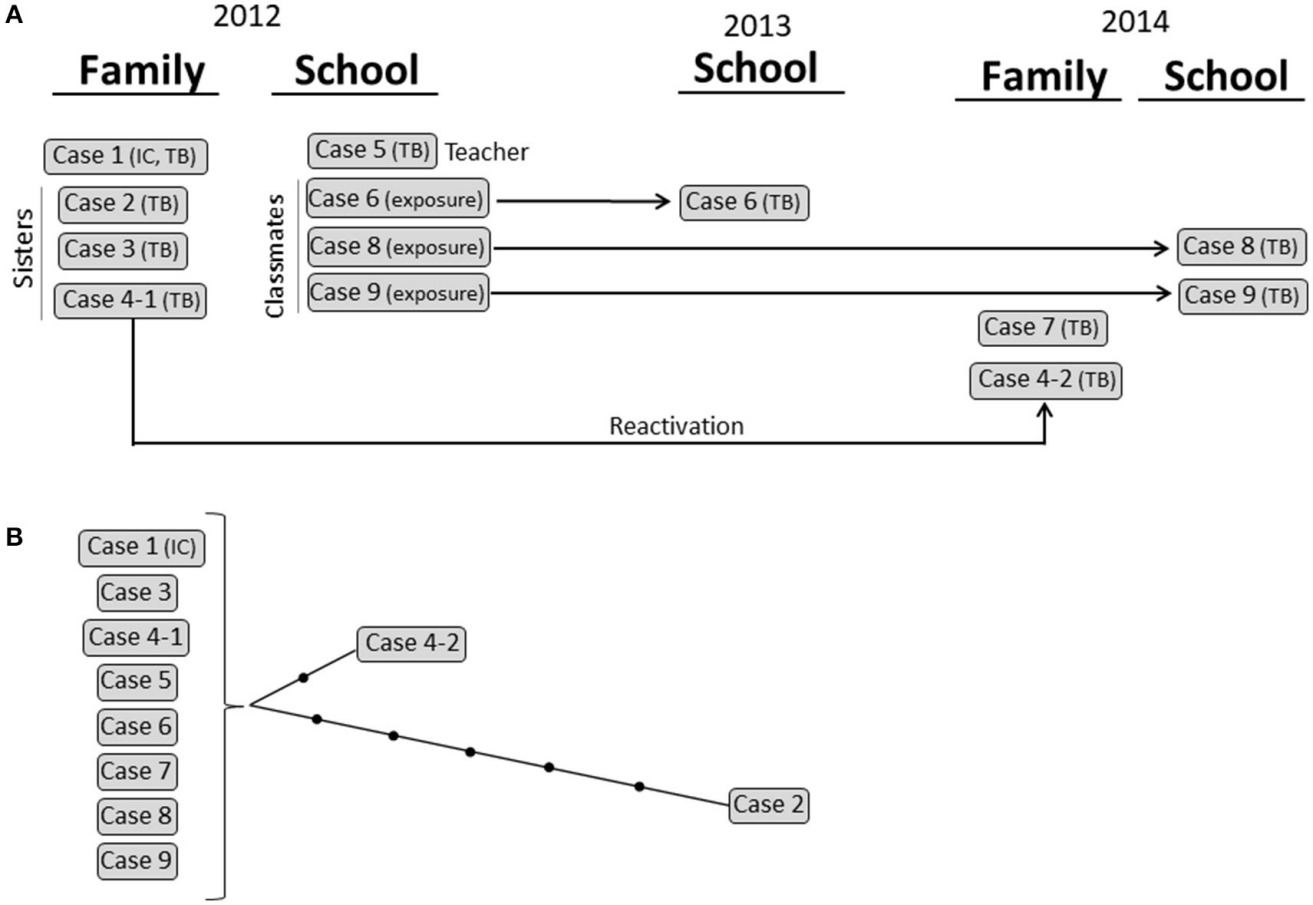
Cluster 3. **(A)** Graphical scheme of the cluster including chronology, transmission settings, and cases involved. **(B)** Network of relationships for the isolates analyzed by WGS. Each black dot corresponds to a SNP. Isolates not available for the WGS analysis are in italic. IC: index case.

Cluster 3 corresponded to a school and household outbreak in Latvia (Figure 4) that involved 5 cases with active TB [1 child (index case), her 3 sisters, and 1 adult (the teacher)] in 2012. Three cases exposed in 2012 developed the disease 1 year later (case 6) and 2 years later (cases 8 and 9). The scenario was further complicated when one of the 2012 cases (case 4) reactivated 2 years later and transmitted TB to her husband (case 7). All 10 isolates were available and shared an identical MIRU-VNTR pattern. WGS analysis revealed that all but 1 of the cases was identical (0 SNPs); however, in the remaining case (case 2), we observed a burst of variability that led to the accumulation of 5 SNPs (Figure 5; Supplementary Table 5).

## Discussion

Microevolution is a phenomenon which can be modulated by various factors. We have to consider it at least a bifactorial event in which both bacterial and host factors intervene. The role of bacterial factors is illustrated by the finding that certain strains from the Beijing lineage show a hypermutator phenotype (Ebrahimi-Rad et al., 2003)- Other non-Beijing strains have been also described to be prone to acquire diversity (Navarro et al., 2017). Microevolution events leading to higher than expected accumulation of variability have been partially addressed in a previous study from our group (Pérez-Lago et al., 2014). Some of these events involved sequential host-to-host transmissions, but an equivalent degree of diversity was observed also within individuals. This intrapatient diversity was detected between isolates infecting different organs, in patients with respiratory and extrarespiratory infection, but also in isolates recovered strictly from the respiratory site. These data mean that the intrapatient diversity might impact on the inference of recent transmission clusters (Pérez-Lago et al., 2014).

As our previous study was based on a convenience sample of isolates selected once they had been shown to have microevolved, we now aimed to perform a more systematic evaluation (i.e., with no preselection of microevolved isolates) of the impact of several circumstances on the acquisition of variability that MTB infection could meet in various clinical or epidemiological scenarios. As with any evolutionary process, microevolution in MTB is the combination of the opportunities the microorganism has to acquire variability and the occurrence of selective pressure bottlenecks that force the selection of clonal variants from among those which emerge. Following this rationale, we selected clinical/epidemiological situations that could lead to increased acquisition of variability (both intrapatient and interpatient).

We began with the intrapatient scenario by selecting single case infections, looking for situations with longer periods of active infection (prolonged infections) in order to increase the likelihood of the emergence of variants and/or looking for factors involved in selective pressure (such as intermittent poor adherence to treatment or transitions from latency to active infection in reactivations). In general, our findings highlight the limited variability acquired, which was within the ranges expected. However, in 1 reactivation (case 6, Supplementary Table 1), we observed greater accumulation of variability (5 SNPs), thus reaching the lower threshold for considering 2 cases as not epidemiologically linked (Walker et al., 2013). Previously, we had also observed intrapatient situations leading to a higher accumulation of SNPs (Pérez-Lago et al., 2014; Navarro et al., 2017).

The non-systematic association between a greater accumulation of variability and clinical situations that could theoretically favor it underlines the multifactorial nature of this phenomenon. Observations from other studies also lead to contradictory findings. Whereas no variability is found in circumstances where it is highly likely, such as long-term persistent infection by a Beijing strain with constant intermittent treatment (Pérez-Lago et al., 2015a), marked variability is detected in less extreme situations (Pérez-Lago et al., 2014; Navarro et al., 2017). It is also true that greater diversity is described in studies involving MDR strains in which a more dynamic process of acquisition of variability is expected owing to constant competition between resistant and compensatory mutations (Merker et al., 2013), although competition between clonal variants is also expected in heterogeneous populations involving susceptible strains.

We then moved on to the interpatient analysis, which focused first on extensive clusters to evaluate the acquisition of variability when 2 key factors coincided, namely, prolonged observation time and adaptation to a high number of sequentially infected hosts. However, the maximum genetic distance accumulated never surpassed 9 SNPs, and the distance between sequential isolates ranged from 1 to 6 SNPs, i.e., within the previously established thresholds although approaching in some cases to their limits (Walker et al., 2013).

We then looked for the coincidence of several of the factors that had been evaluated independently, making it possible to observe amplification of effects that could only have a subtle impact when evaluated one by one. It was not easy to find transmission events with the intervention of as many as possible of these factors, namely, extended time to allow microevolution to occur, involvement of a high number of hosts to ensure sequential selective pressures due to the establishment of each infection in different hosts, and overlap of transitions from latency to active infections. These kind of clusters with such a complex epidemiological peculiarities were insufficiently represented in the studies defining the SNP thresholds to be applied as a reference in genomic epidemiology (Walker et al., 2013). We found 3 clusters fulfilling all or most of our requirements, each from a different population and involving 2 countries. However, despite the greater opportunities to acquire diversity that the clinical/epidemiological situations in these complex clusters offered, the global variability identified generally lay within the thresholds established, which limits the impact of this diversity on the inference of recent transmission in genomic epidemiology studies.

Although our main aim was to perform a quantitative analysis of the number of SNPs acquired, we had the opportunity to record interesting qualitative observations. The first was the finding that some SNPs (1 each in case 4 and case 6, Supplementary Table 1; and one each in case 1 and 4-1 in cluster 2, Supplementary Table 5) were only observed in the first isolate of a chronological series. While this observation may at first sight seem incongruent, owing to the negligible degree of homoplasy described for *Mycobacterium tuberculosis complex* (Hershberg et al., 2008; Comas et al., 2009) and the highly unlikely reversion of mutations once acquired, we believe that it might be explained by undetected coexistence of the variant which acquired the SNP together with the parental variant, which did not have the SNP. Given the unavoidable limitations of sputum sampling with respect to the whole bacterial population in the lung, we likely only detected 1 of the coexisting variants at each sampling point. The observation that isolates recovered from 1 sputum sample do not represent true clonal complexity in the lung has been addressed elsewhere (Black et al., 2015; Pérez-Lago et al., 2015b).

About the functional significance of SNPs (13 synonymous and 40 non-synonymous SNPs), it must be highlighted a lower nonsynonymous/synonimous ratio (0.325) compared with a global database including 220 strains (0.606, *p* = 0.04 Fisher's exact test). The difference is mainly driven by the accumulation of a lower number of nonsynonymous changes in intrapatient cases compared to inter-patient transmission cluster (dN/dS 0.353 vs. 1.714). This observation is in contrast with the expectation that shorter timespan should be associated to higher dN/dS ratios. The lower number of nonysnonymous changes intrapatient may indicate the action of purifying selection. Purifying selection is expected during treatment course (Trauner et al., 2017) and can remove diversity faster, given the clonal nature of MTBC populations. However, the heterogeneity of the dataset involving different patients and transmission clusters as well as the low number of mutations prevents us to support any general conclusion.

Our global analysis did not enable us to identify a clinical/epidemiological situation that could predict greater accumulation of diversity in MTB. We must admit certain limitations resulting of not having considered the diversity based on structural variants and indels or not having performed a high-depth analysis or minority variants. We have focused on diversity based on SNPs mainly due to the fact that this is the criterion in which genomic epidemiology lies, and thus to evaluate the impact of this variability on the inference of recent transmission. The number of SNPs identified under circumstances that could theoretically favor microevolution was limited, within the ranges expected according to the proposed similarity thresholds for considering 2 isolates as related or unrelated (Walker et al., 2013). These findings support the robustness of the cutoffs. However, we also found asymmetric bursts of variability, as reported elsewhere (Pérez-Lago et al., 2014; Navarro et al., 2017). The coincidence of several such cases in the same transmission event could eventually lead us to overlook some transmission clusters.

We must also accept that despite trying to force the coincidence of factors theoretically favoring microevolution, some of these factors, such as length of the active infection period, serial involvement of sequentially infected hosts due to active recent transmission, and lack of adherence to treatment, could be more frequent and of a greater magnitude in settings with weaker diagnostic and control programs. Studies similar to ours should be replicated in these settings to enrich our knowledge of the circumstances which could favor diversity in TB and to determine more precisely its impact on the tracking of transmission.

## Author contributions

Experimental load: all authors; Writing: LP-L, DG-d-V; Design conceptualization: LP-L, DG-d-V; Bioinformatic Analysis and design: ÁC-O, IC.

### Conflict of interest statement

The authors declare that the research was conducted in the absence of any commercial or financial relationships that could be construed as a potential conflict of interest.

## Supplementary material

Supplementary Table 1 SNPs and features for the SNPs found for the intrapatient analysis.

Supplementary Table 2 SNPs and features for the SNPs found for the Cluster F.

Supplementary Table 3 SNPs and features for the SNPs found for the Cluster 6.

Supplementary Table 4 SNPs and features for the SNPs found for the Cluster B.

Supplementary Table 5 SNPs and features for the SNPs found for the Clusters 1, 2 and 3.
