# Supplementary material for: Mycobacterium tuberculosis Acquires Limited Genetic Diversity in Prolonged Infections, Reactivations and Transmissions Involving Multiple Hosts
Source: Front Microbiol. 2018 Jan 19;8:2661. doi: 10.3389/fmicb.2017.02661 (PMC5780704; doi:10.3389/fmicb.2017.02661)
Supplement: Supplementary Table 1 — SNPs and features for the SNPs found for the intrapatient analysis. [file Table1.pdf]

Supplementary table 1

Prolonged Infections

Case 1

20 months							
**1st isolate (1996)**	**2nd isolate (1998)**	**ANC**	**Change**	**Essentiality Prediction**	**Position**	**Gene**	**Function**
G	**A**	G	Intergenic	NA	3841685	Rv3423-Rv3424	NA
G	**A**	G	Intergenic	NA	3846613	Rv3428-Rv3429	NA

Case 2

20 months							
**1st isolate (1996)**	**2nd isolate (1998)**	**ANC**	**Change**	**Essentiality Prediction**	**Position**	**Gene**	**Function**
A	**G**	A	Nonsynonymous (Lys/Glu)	essential	3008390	Rv2690c	Unknown

Case 3

22 months							
**1st isolate (1995)**	**2nd isolate (1997)**	**ANC**	**Change**	**Essentiality Prediction**	**Position**	**Gene**	**Function**
C	**T**	C	Nonsynonymous (Ala/Ser)	Essential	3008024	Rv2690c	Unknown

Case 4

29 months							
**1st isolate (1996)**	**2nd isolate (1999)**	**ANC**	**Change**	**Essentiality Prediction**	**Position**	**Gene**	**Function**
**A**	G	G	Synonymous	Essential	2686795	Rv2392	Sulfate activation pathway cysH
G	**A**	G	Nonsynonymous (Thr a/Iso)	Non essential	1075104	Rv0962	Possible lipoprotein LprP
G	**C**	G	Synonymous	Non essential	2534929	Rv2262c	Unknown
T	**G**	T	Intergenic		2692188	Rv2395-Rv2396	NA

Reactivations

Case 5

27 months							
**1st isolate (2003)**	**2nd isolate (2005)**	**ANC**	**Change**	**Essentiality Prediction**	**Position**	**Gene**	**Function**
A	**C**	A	Nonsynonymous (Leu a Pro)	Non essential	549561	Rv0457c	Probable peptidase

Case 6

56 months							
**1st isolate (1998)**	**2nd isolate (2002)**	**ANC**	**Change**	**Essentiality Prediction**	**Position**	**Gene**	**Function**
C	**T**	C	Synonymous	Non essential	144862	Rv0119	Involved in lipid degradation fadD7
T	**C**	T	Synonymous	Non essential	794435	Rv0694	Involved in respiration lldD1
G	**C**	G	Synonymous	Essential	1921712	Rv1697	Unknown
C	**T**	C	Nonsynonymous (Val a Met)	Non essential	2564843	Rv2293c	Unknown
C	**G**	C	Nonsynonymous (Arg a Pro)	Non essential	3330490	Rv2974c	Unknown
**C**	T	T	Synonymous	Essential	4168012	Rv3722c	Unknown

SNPs are labelled in bold and underlined; ANC: ancestor alelle; NA: not applicable
