# Supplementary material for: Mycobacterium tuberculosis Acquires Limited Genetic Diversity in Prolonged Infections, Reactivations and Transmissions Involving Multiple Hosts
Source: Front Microbiol. 2018 Jan 19;8:2661. doi: 10.3389/fmicb.2017.02661 (PMC5780704; doi:10.3389/fmicb.2017.02661)
Supplement: Supplementary Table 2 — SNPs and features for the SNPs found for the Cluster F. [file Table2.pdf]

Supplementary Table 2

Cluster F															
Cases															
**A (2007)**	**B (2007)**	**C (2007)**	**D (2008)**	**E (2008)**	**F (2008)**	**G (2012)**	**H (2012)**	**I (2013)**	**ANC**	**Change**	**Essentiality prediction**	**Position**	**Gene**	**Function**
C	C	C	**G**	C	C	C	C	C	C	Non-synonymous (Ala/Gly)	Essential	604458	Rv0511	Probable uroporphyrin-III C-methyltransferase HemD
A	**G**	A	A	A	A	A	A	A	A	Non-synonymous (Asp/Gly)	Non essential	643979	Rv0522	Unknown
**T**	C	C	C	C	C	C	C	C	C	Non-synonymous (Arg/Gln)	Non essential	705812	Rv0610	Unknown
C	C	C	C	**T**	C	C	C	C	C	Synonymous	Essential	905762	Rv0811	Unknown
T	T	T	T	**C**	T	T	T	T	T	Non-synonymous (Thr/Ala)	Non essential	1134211	Rv1015	50S ribosomal protein L25 RplY
**T**	C	C	C	C	C	C	C	C	C	Non-synonymous (Val/Iso)	Non essential	1436334	Rv1283	Probable oligopeptide-transport membrane prot OppB
T	T	T	T	T	T	T	T	**C**	T	Non-synonymous (Val/Ala)	Non essential	1839937	Rv1634	Possible drug efflux memb prot
**C**	T	T	T	T	T	T	T	T	T	Intergenic	NA	2489172	Rv2220-Rv2221	NA
**C**	A	A	A	A	A	A	A	A	A	Non-synonymous (Tyr/Asp)	Essential	3350034	Rv2992	Glutamyl-tRNA synthetase GltS
G	G	G	**A**	G	G	G	G	G	G	Non-synonymous (Gly/Asp)	Non essential	3512776	Rv3147	Probable NADH dehydrogenase I NuoC
C	**T**	C	C	C	C	C	C	C	C	Non-synonymous (Gln/STOP)	Non essential	3805207	Rv3390	Probable conserved lipoprotein LpqD

ANC: ancestor; NA: not applicable ; SNPs are labelled in bold and underlined
