# Supplementary material for: Mycobacterium tuberculosis Acquires Limited Genetic Diversity in Prolonged Infections, Reactivations and Transmissions Involving Multiple Hosts
Source: Front Microbiol. 2018 Jan 19;8:2661. doi: 10.3389/fmicb.2017.02661 (PMC5780704; doi:10.3389/fmicb.2017.02661)
Supplement: Supplementary Table 3 — SNPs and features for the SNPs found for the Cluster 6. [file Table3.pdf]

Supplementary Table 3

Cluster 6																						
Cases																						
**A (2003)**	**B (2004)**	**C (2004)**	**D (2005)**	**E (2005)**	**F (2006)**	**G (2006)**	**H (2006)**	**I (2006)**	**J (2007)**	**K (2007)**	**L (2009)**	**M (2009)**	**N (2009)**	**O (2010)**	**P (2013)**	**Q (2014)**	**ANC**	**Change**	**Esssentiality prediction**	**Position**	**Gene**	**Function**
C	C	C	C	C	C	C	**T**	C	C	C	C	C	C	C	C	C	C	Non-synonymous (Ala/Val)	Essential	8452	Rv0006	DNA gyrase (subunit A) GyrA
C	C	C	C	C	C	C	C	C	C	C	C	C	C	**T**	C	C	C	Non-synonymous (Pro/Ser)	not tested	10046	Rv0007	Unknown
T	T	T	T	T	T	**C**	T	**C**	T	T	T	T	T	T	T	T	T	Non-synonymous (Met/Val)	Non essential	51391	Rv0047	Unknown
**G**	A	A	**G**	**G**	A	A	**G**	A	**G**	**G**	**G**	**G**	**G**	**G**	A	**G**	A	Non-synonymous (Asp/Gly)	Non essential	116437	Rv0101	Probable peptide synthetase Nrp
C	C	C	C	C	C	**T**	C	C	C	C	C	C	C	C	C	C	C	Intergenic		580806	Rv0490-Rv0491	
A	A	A	A	**C**	A	A	A	A	A	A	A	A	A	A	A	A	A	Non-synonymous (Asp/Ala)	Non essential	1139505	Rv1020	Probable transcription-repair coupling factor Mfd
C	C	C	C	C	**A**	C	C	C	C	C	C	C	C	C	C	C	C	Non-synonymous (Ala/Glu)	Non essential	1620683	Rv1441	Probable biotin sulfoxide reductase BisC
T	C	C	C	C	C	C	C	C	C	C	C	C	C	C	**T**	C	C	Non-synonymous (Ala/Val)	Essential	1939070	Rv1711	Unknown
C	C	C	C	C	C	C	C	C	C	C	C	**T**	C	C	C	C	C	Non-synonymous (Gly/Arg)	Non essential	1977804	Rv1749	Unknown
**C**	A	A	**C**	**C**	**C**	A	**C**	A	**C**	**C**	**C**	**C**	**C**	**C**	A	**C**	A	Non-synonymous (Asn/Thr)	not tested	2217534	Rv1973	Unknown
**C**	G	G	**C**	**C**	G	G	**C**	G	**C**	**C**	**C**	**C**	**C**	**C**	G	**C**	G	Non-synonymous (Pro/Ala)	Non essential	2787288	Rv2482	Probable glycerol-3-phosphate acyltransferase
**G**	G	G	G	G	**A**	G	G	G	G	G	G	G	G	G	G	G	G	Non-synonymous (Thr/Met)	Non essential	2792367	Rv2484	Possible triacylglycerol synthase
A	A	A	A	A	A	A	**C**	A	A	A	A	A	A	A	A	A	A	Non-synonymous (Leu/Arg)	Non essential	2902750	Rv2578	Unknown
A	A	A	A	A	A	**C**	A	**C**	A	A	A	A	A	A	A	A	A	Non-synonymous (Ser/Ala)	Essential	2939047	Rv2611	Probable acyltransferase
G	G	G	G	G	**A**	G	G	G	G	G	G	G	G	G	G	G	G	Synonymous	Essential	2950201	Rv2623	Universal stress protein family protein TB31.7
A	A	A	A	**G**	A	A	A	A	A	A	A	A	A	A	A	A	A	Intergenic		3486475	Rv3120-Rv3121	
G	G	G	G	G	G	G	G	G	**A**	G	G	G	G	G	G	G	G	Non-synonymous (Asp/Asn)	Essential	3728145	Rv3341	Probable homoserine O-acetyltransferase MetA
G	G	G	G	G	G	G	G	G	G	G	G	G	G	G	**A**	G	G	Synonymous	Essential	3813584	Rv3396	Probable GMP synthase GuaA
T	**G**	**G**	T	T	T	T	T	T	T	T	T	T	T	T	**G**	T	T	Non-synonymous (Asp/Ala)	Non essential	4118099	Rv3678	Unknown
G	**A**	**A**	G	G	G	G	G	G	G	G	G	G	G	G	**A**	G	G	Non-synonymous (Ala/Thr)	Non essential	4226398	Rv3779	Unknown

ANC: ancestor; NA: not applicable ; SNPs are labelled in bold and underlined
