# Supplementary material for: Mycobacterium tuberculosis Acquires Limited Genetic Diversity in Prolonged Infections, Reactivations and Transmissions Involving Multiple Hosts
Source: Front Microbiol. 2018 Jan 19;8:2661. doi: 10.3389/fmicb.2017.02661 (PMC5780704; doi:10.3389/fmicb.2017.02661)
Supplement: Supplementary Table 4 — SNPs and features for the SNPs found for the Cluster B. [file Table4.pdf]

Supplementary Table 4

Cluster B																	
Cases																	
**A (2003)**	**B (2005)**	**C (2006)**	**D (2007)**	**E (2007)**	**F (2008)**	**G (2008)**	**H (2008)**	**I (2008)**	**J (2011)**	**K (2011)**	**L (2015)**	**ANC**	**Change**	**Essentiality prediction**	**Position**	**Gene**	**Function**
C	C	C	C	C	C	C	C	C	**<u>T</u>**	C	C	C	Synonymous	non essential	964612	Rv0867	Possible resuscitation-promoting factor RpfA
T	T	T	T	T	T	T	T	T	**<u>G</u>**	T	T	T	Non-synonymous (Glu/Asp)	non essential	964618	Rv0867	Possible resuscitation-promoting factor RpfA
G	G	G	G	G	G	G	G	G	**<u>T</u>**	G	G	G	Non-synonymous (Asp/Glu)	non essential	964642	Rv0867	Possible resuscitation-promoting factor RpfA
G	G	G	G	G	G	G	G	**<u>T</u>**	G	**<u>T</u>**	G	G	Synonymous	non essential	1103408	Rv0987	Probable adhesion component transport transmemb prot
G	G	G	G	G	**<u>T</u>**	G	G	G	G	G	G	G	Intergenic	NA	1426155	Rv1276-Rv1277	NA
T	T	T	T	T	T	T	T	**<u>C</u>**	T	**<u>C</u>**	T	T	Non-synonymous (Thr/Ala)	non essential	2081726	Rv1835	Unknown
T	T	T	T	T	T	T	**<u>C</u>** /T	T	T	T	**<u>C</u>**	T	Synonymous	non essential	2301782	Rv2048	Polyketide synthase Pks12
G	G	G	G	G	G	G	**<u>A</u>**	G	G	G	**<u>A</u>**	G	Non-synonymous (Arg/Cys)	non essential	2302033	Rv2048	Polyketide synthase Pks12
A	A	A	A	A	**<u>C</u>**	A	A	A	A	A	A	A	Synonymous	non essential	2389305	Rv2127	L-asparagine permease AnsP1
C	C	C	C	C	C	C	C	C	C	C	**<u>A</u>**	C	Intergenic	NA	2516698	Rv2242-Rv2243	NA
T	T	T	T	T	T	**<u>C</u>**	T	T	T	T	T	T	Non-synonymous (Trp/Arg)	non essential	2687818	Rv2393	Ferrochelatase Che1
A	A	A	A	A	A	A	A	**<u>G</u>**	A	**<u>G</u>**	A	A	Non-synonymous (Phe/Ser)	essential	3214081	Rv2904	50S ribosomal protein L19 RplS
**<u>C</u>**	T	T	T	T	T	T	T	T	T	T	T	T	Non-synonymous (Iso/Val)	non essential	3880351	Rv3461	50S ribosomal protein L36 RpmJ
C	**<u>A</u>**	C	C	C	C	C	C	C	C	C	C	C	Non-synonymous (Glu/STOP)	non essential	3890622	Rv3473	Possible peroxidase BpoA
A	A	A	A	A	A	A	A	A	A	A	**<u>G</u>**	A	Non-synonymous (Val/Ala)	non essential	3893506	Rv3476	Probable dicarboxylic acid transport integral memb prot KgtP

ANC: ancestor; NA: not applicable ; SNPs are labelled in bold and underlined
