# Supplementary material for: Mycobacterium tuberculosis Acquires Limited Genetic Diversity in Prolonged Infections, Reactivations and Transmissions Involving Multiple Hosts
Source: Front Microbiol. 2018 Jan 19;8:2661. doi: 10.3389/fmicb.2017.02661 (PMC5780704; doi:10.3389/fmicb.2017.02661)
Supplement: Supplementary Table 5 — SNPs and features for the SNPs found for the Clusters 1, 2 and 3. [file Table5.pdf]

Supplementary Table 5

Cluster 1

Case 1 (2012)	Case 3.I (2015)	Case 3.II (2015)	Case 3.III (2015)	Case 3.IV (2015)	Case 4 (2015)	Case 5 (2015)	Case 6 (2015)	Case 7 (2015)	Case 8 (2015)	Case 9 (2015)	ANC	Change	Essentiality prediction	Position	Gene	Function
G	<u>**C**</u>	<u>**C**</u>	<u>**C**</u>	<u>**C**</u>	<u>**C**</u>	<u>**C**</u>	<u>**C**</u>	<u>**C**</u>	<u>**C**</u>	<u>**C**</u>	G	Non-synonymous (Gly/Ala)	Non essential	66129	Rv0062	Cellulase celA1
A	A	A	A	A	A	A	A	A	A	<u>**G**</u>	A	Intergenic	NA	2310789	Rv2051-Rv2052	NA
G	G	G	G	G	G	G	G	G	G	<u>**C**</u>	G	Non-synonymous (Thr/Arg)	Essential	3135912	Rv2828	Unnknown

ANC: ancestor; NA: not applicable; SNPs are labeled in bold and underlined

Cluster 2

Case 1 (2009)	Case 3 (2011)	Case 4-1 (2012)	Case 5 (2013)	Case 6-1 (2013)	Case 2-2 (2015)	Case 4-2 (2015)	ANC	Change	Essentiality prediction	Position	Gene	Function
G	G	G	G	G	<u>**A**</u>	G	G	Non-synonymous (Gly/Ser)	Non essential	1332179	Rv1189	Possible alternative RNA polymerase sigma factor SigI
A	A	A	A	<u>**G**</u>	A	A	A	Non-synonymous (Iso/Val)	Essential	3506764	Rv3139	Possible acyl-coA dehydrogenase fadE24
<u>**T**</u>	C	C	C	C	C	C	C	Synonymous	Non essential	832087	Rv0740	Unknown
C	C	<u>**T**</u>	C	C	C	C	C	Synonymous	Non essential	2135571	Rv1886	Involved in cell wall mycoloylation fbPB
C	C	C	C	C	<u>**T**</u>	C	C	Synonymous	Non essential	3599414	Rv3223	Alternative sigma factor SigH

ANC: ancestor; SNPs are labeled in bold and underlined

Cluster 3

Case 1 (2012)	Case 2 (2012)	Case 3 (2012)	Case 4-1 (2012)	Case 5 (2012)	Case 6 (2013)	Case 7 (2014)	Case 8 (2014)	Case 9 (2014)	Case 4-2 (2014)	ANC	Change	Essentiality prediction	Position	Gene	Function
T	<u>**C**</u>	T	T	T	T	T	T	T	T	T	Non-synonymous (Glu/Gly)	Non essential	182945	Rv 0154	Probable acyl-coA dehydrogenase fadE2
C	<u>**T**</u>	C	C	C	C	C	C	C	C	C	Synonymous	Non essential	201744	Rv0171	Involved in host cell invasion mce1C
T	<u>**C**</u>	T	T	T	T	T	T	T	T	T	Non-synonymous (Trp/Arg)	Non essential	2097084	Rv1847	Unknown
C	<u>**T**</u>	C	C	C	C	C	C	C	C	C	Non-synonymous (Asp/Asn)	Non essential	2143843	Rv1896	Unknown
C	<u>**T**</u>	C	C	C	C	C	C	C	C	C	Synonymous	Non essential	3768865	Rv3353	Unknown
G	G	G	G	G	G	G	G	G	<u>**A**</u>	G	Non-synonymous (Ser/Leu)	Non essential	4365619	Rv3884	ESX conserved component EccA2

ANC: ancestor; SNPs are labeled in bold and underlined
