# Supplementary material for: Mycobacterium tuberculosis Acquires Limited Genetic Diversity in Prolonged Infections, Reactivations and Transmissions Involving Multiple Hosts
Source: Front Microbiol. 2018 Jan 19;8:2661. doi: 10.3389/fmicb.2017.02661 (PMC5780704; doi:10.3389/fmicb.2017.02661)
Supplement: Supplementary file 6 [file DataSheet1.pdf]

List of the correlation between isolate names and accession numbers of project PRJEB1553

ClusterB-caseB	ERS737782
ClusterB-caseF	ERS737783
ClusterB-caseG	ERS737784
ClusterB-caseH	ERS737787
ClusterF-caseA	ERS737789
ClusterF-caseB	ERS737815
ClusterF-caseD	ERS737814
